# Differences of white matter structure for diffusion kurtosis imaging using voxel-based morphometry and connectivity analysis

**DOI:** 10.1093/bjro/tzad003

**Published:** 2023-12-12

**Authors:** Yuki Kanazawa, Natsuki Ikemitsu, Yuki Kinjo, Masafumi Harada, Hiroaki Hayashi, Yo Taniguchi, Kosuke Ito, Yoshitaka Bito, Yuki Matsumoto, Akihiro Haga

**Affiliations:** Graduate School of Biomedical Sciences, Tokushima University, Tokushima 770-8503, Japan; Division of Radiological Technology, Okayama University Hospital, Okayama 700-8558, Japan; Department of Radiology, Higashihiroshima Medical Center, National Hospital Organization, Hiroshima 739-0041, Japan; Graduate School of Biomedical Sciences, Tokushima University, Tokushima 770-8503, Japan; College of Medical, Pharmaceutical and Health Sciences, Kanazawa University, Ishikawa 920-0942, Japan; FUJIFILM Healthcare Corporation, Tokyo 107-0052, Japan; FUJIFILM Healthcare Corporation, Tokyo 107-0052, Japan; FUJIFILM Healthcare Corporation, Tokyo 107-0052, Japan; Graduate School of Biomedical Sciences, Tokushima University, Tokushima 770-8503, Japan; Graduate School of Biomedical Sciences, Tokushima University, Tokushima 770-8503, Japan

**Keywords:** magnetic resonance imaging, diffusion kurtosis, white matter, voxel-based morphometry, diffusion tensor tractography

## Abstract

**Objectives:**

In a clinical study, diffusion kurtosis imaging (DKI) has been used to visualize and distinguish white matter (WM) structures’ details. The purpose of our study is to evaluate and compare the diffusion tensor imaging (DTI) and DKI parameter values to obtain WM structure differences of healthy subjects.

**Methods:**

Thirteen healthy volunteers (mean age, 25.2 years) were examined in this study. On a 3-T MRI system, diffusion dataset for DKI was acquired using an echo-planner imaging sequence, and T_1_-weghted (T_1_w) images were acquired. Imaging analysis was performed using Functional MRI of the brain Software Library (FSL). First, registration analysis was performed using the T_1_w of each subject to MNI152. Second, DTI (eg, fractional anisotropy [FA] and each diffusivity) and DKI (eg, mean kurtosis [MK], radial kurtosis [RK], and axial kurtosis [AK]) datasets were applied to above computed spline coefficients and affine matrices. Each DTI and DKI parameter value for WM areas was compared. Finally, tract-based spatial statistics (TBSS) analysis was performed using each parameter.

**Results:**

The relationship between FA and kurtosis parameters (MK, RK, and AK) for WM areas had a strong positive correlation (FA-MK, *R*^2^ = 0.93; FA-RK, *R*^2^ = 0.89) and a strong negative correlation (FA-AK, *R*^2^ = 0.92). When comparing a TBSS connection, we found that this could be observed more clearly in MK than in RK and FA.

**Conclusions:**

WM analysis with DKI enable us to obtain more detailed information for connectivity between nerve structures.

**Advances in knowledge:**

Quantitative indices of neurological diseases were determined using segmenting WM regions using voxel-based morphometry processing of DKI images.

## Introduction

The white matter (WM) is a part of the cerebral hemisphere where nerve cell bodies are scarce and axons extend and accumulate from nerve cell bodies in the grey matter (GM), running nerve fibre bundles throughout. The diffusion tensor imaging (DTI) method can noninvasively evaluate the neural structure of the brain, which captures the anisotropy of water molecule diffusion using diffusion-weighted imaging (DWI).^1^ This method calculates the apparent diffusion coefficient and fractional anisotropy (FA), which represents the degree of diffusion anisotropy. Bulk water diffusion of DTI analysis has been generally assumed to have a normal distribution. However, actual *in vivo* microstructures are complex, and therefore, it is difficult to derive a normal distribution. To improve an analysis procedure, diffusion kurtosis imaging (DKI) has been developed, which does not depend on the normal distribution and does not assume a biophysical model; it became an alternative evaluation method.^2–4^ In addition, there are other diffusion models, for example, neurite orientation dispersion and density imaging^5^ and mean apparent propagator.^6^ The usefulness and potential of the DTI method to diagnose brain tumours, demyelinating diseases, and degenerative diseases have been reported elesewhere.^7^

The DTI neural analysis method includes FA mapping and diffusion tensor tractography (DTT) procedures, which are unique tools for the noninvasive characterization of tissue microstructural properties. The FA value is an index of anisotropic diffusion. When the FA value approaches "zero," it is close to isotropic diffusion. When the value approaches “one,” it is close to anisotropic diffusion. An FA map is an image of the magnitude of the FA value. WM is rich myelinated nerve fibres in the central nervous system, and therefore, it has high diffusion anisotropy. In contrast, DTT has a 3-dimensional macroscopic structure consisting of continuous nerve fibre pathways according to FA map information.^8^ Voxel-based morphometry (VBM) is a method to analyse brain volume, which can segment brain tissue (WM, GM, etc.) automatically and evaluate the whole brain objectively.^9^ This method has been applied to psychiatric and neurological disorders associated with brain atrophy. Tract-based spatial statistics (TBSS) utilizes the following fact; a slight FA change can be observed in the direction being parallel to the nerve fibre and the change becomes greater in the perpendicular direction.^10^ This phenomenon allows us to create a cylindrical and/or sheet-like skeletal structure from the FA maps for all subjects. Using this skeletal structure, the FA map is normalized and then analysed.^11^

Parameter images calculated from DKI are classified into the following categories; axial diffusivity (AD): parallel to the main axis of the diffusion tensor, radial diffusivity (RD): perpendicular to the main axis, and the mean diffusivity (MD) in all axial directions, mean kurtosis (MK), axial kurtosis (AK), and radial kurtosis (RK) as deviations.^12^ Since DKI is considered to be consistent with the structure of nerve fibre bundles and the deviations of heterodirectional diffusion in voxels, there have been many studies related to WM tracts using DKI.^13^ Additionally, numerical comparisons of normal WM regions based on DKI analysis and the intersections of nerve fibre runs, such as WM tracts, have been reported.^4^^,^^14^ However, there has been no numerical comparison of DKI in various regions of normal WM, and no verification of the connections at the intersections of nerve fibre extensions, that is, regions of high nerve density. Multiple sclerosis (MS) is a demyelinating disease in which the myelin sheaths protecting the nerves are damaged, and abnormalities are often detected in areas of high nerve density.^15^ When evaluating pathologies such as MS using DKI and DTI, it is important to understand the normal differences between each parameter for each WM region. The objective of our study is to evaluate and compare the DTI and DKI parameters based on VBM to obtain WM structure differences of healthy subjects. In addition, we assessed the connections between the DTI and DKI nerve fibre pathways with TBSS.

## Methods

### MR imaging

This study was performed between November 2019 and January 2021. This study was approved by the Internal Review Board. Healthy subjects were recruited in our institute, from whom written informed consent was obtained prior to participation. All subjects were proven without abnormal signals by a neuroradiologist. The exclusion criteria were imaging data with heavy artefacts and abnormal signals: all subjects were out of the criteria. The 13 subjects (10 males and 3 females; age range, 21-43 years; mean age, 25.2 years) were scanned using a 3-T MRI system (FUJIFILM Healthcare Corporation, Tokyo, Japan). The DKI datasets were obtained using echo-planner imaging (EPI). The imaging parameters were echo time (TE), 110 ms; repetition time (TR), 6500 ms; flip angle (FA), 90°; matrix size, 128 × 128 × 72; voxel size, 1.88 × 1.88 × 2 mm^3^; field of view (FOV), 240 × 240 mm^3^. The DTI and DKI parameter images were acquired from EPI images with multiple *b*-values (0, 800, and 2500 s/mm^2^) and 21-diffusion gradient directions. Then, parallel imaging techniques were applied (acceleration factor of phase direction 2.0) on the 32-channel phased-array head coil. In this experiment, FA was calculated using DTI parameters, and MK, AK, and RK were calculated from DKI parameters. These images were generated from the software incorporated into the MR system,^16^ which can be negligible differences in magnetic field strength.

In addition, RF-spoiled steady-state gradient-echo (RSSG) was utilized to standardize anatomical structures using T_1_-weghted (T_1_w) images. The imaging parameters were set as follows: TE, 6.2 ms; TR, 12 ms; FA, 18°; matrix size, 512 × 512 × 144; voxel size, 0.47 × 0.47 × 1 mm^3^; FOV, 240 × 240 × 144 mm^3^; slice thickness, 2 mm.

### Image analysis

**Figure 1.**
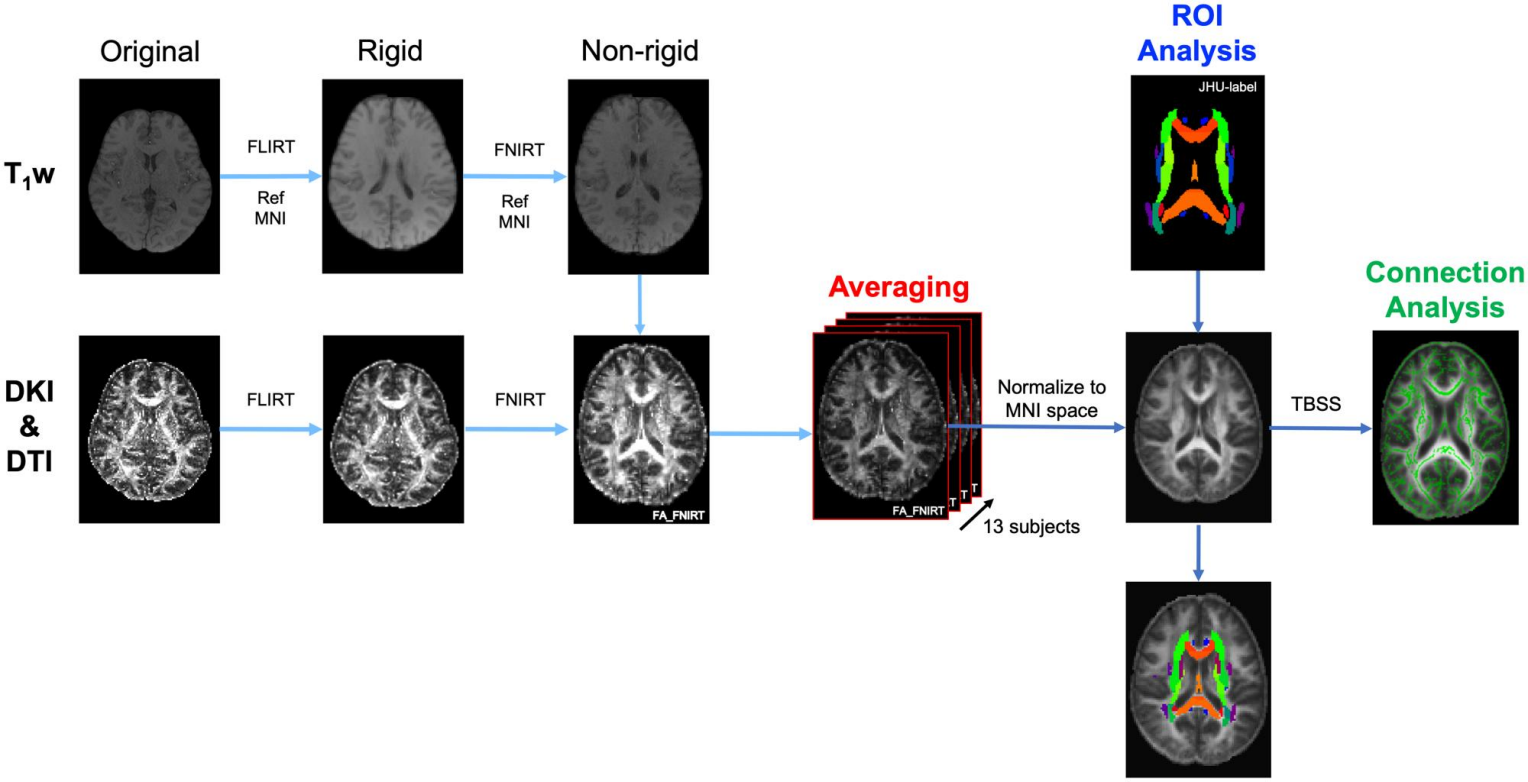
Block diagram of the imaging analysis procedure. The image analysis procedure is shown below: T_1_w is used as an MNI reference image for rigid and nonrigid body alignment. For calculating each parameter image, T_1_w is used as the reference image for rigid and nonrigid alignment. MNI, Montreal Neurological Institute.

Figure 1 shows a schematic drawing of our experimental procedure. VBM processing was performed on each parameter image acquired from DTI and DKI. Before VBM analysis, skull stripping using FSL’s brain extraction tool^17^ was applied to b0 images (DWI with *b*-value 0 s/mm^2^), and the resulting binary masks were applied to DTI and DKI maps. The T_1_w obtained from the RSSG used for standardization was aligned nonlinearly and linearly using the Montreal Neurological Institute (MNI) space as a reference.^18^^,^^19^ In addition, linear and nonlinear alignment was performed with FMRIB's Integrated Registration and Segmentation Tool (FLIRT) and the nonrigid images with FMRIB's Nonlinear Image Registration Tool (FNIRT) for each parameter image using the T_1_w images as a reference.^20^^,^^21^ The spline coefficients and affine matrices obtained were applied to each parameter image to standardize to MNI. This analysis was performed on all thirteen participants. Calculated parameter images were then averaged, and region of interest (ROI) analysis was performed. The ROIs were set using a JHU-WhiteMatter-labels-1mm template.^22^ Mean parameter maps were multiplied with a WM mask that was generated from referenced T_1_w images.

### Statistical analysis

To compare the results between parameters of WM regions of healthy volunteers, the regression analysis between DTI and DKI parameters was performed. The coefficient of determination (*R*^2^) for the regression curve between the DTI and DKI parameters was derived, in which a *P*-value of <0.05 was considered statistically significant. The analysis regions were genu of corpus callosum (GCC), subcallosal cingulate (SCC), anterior limb of the internal capsule (ALIC), posterior limb of the internal capsule (PLIC), retrolenticular part of the internal capsule (RLIC), anterior corona radiata (ACR), posterior thalamic radiation (PTR), and external capsule (EC). Here, no obvious regional biases were found due to the VBM processing for the total subjects. These regions were selected to compare with each parameter value in the previous literature.^23^ In addition, the connections between the DTI and DKI nerve fibre pathways were compared using TBSS.

## Results

**Figure 2.**
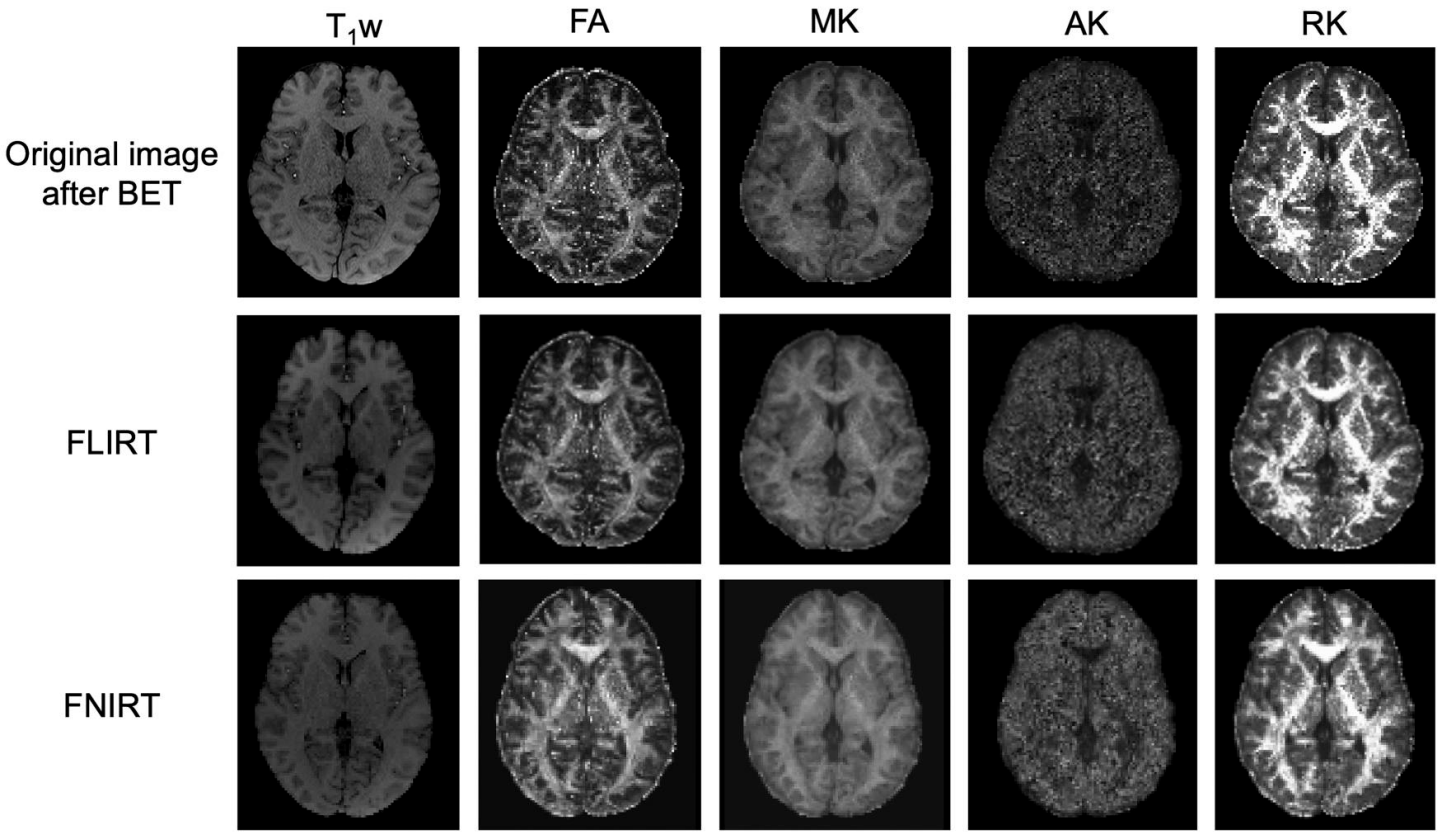
The original, the rigid, and nonrigid aligned images to T_1_w and each parameter image (FA, MK, AK, RK) using FSL software. Top raw images show original image, middle images show the rigid images aquired using FLIRT, and under images show the nonrigid images using FNIRT. Left to right show T_1_w, FA, MK, AK, and RK images, respectively. AK, axial kurtosis; FA, fractional anisotropy; FNIRT, FMRIB's Nonlinear Image Registration Tool; MK, mean kurtosis; RK, radial kurtosis; T_1_w, T_1_ weghted.

Figure 2 shows original, the rigid, and nonrigid aligned images to T_1_w and each parameter image (FA, MK, AK, and RK) using FSL software. The rigid image was acquired with FLIRT and FNIRT. Each image was registered to MNI space after rigid and nonrigid transformation.

**Figure 3.**
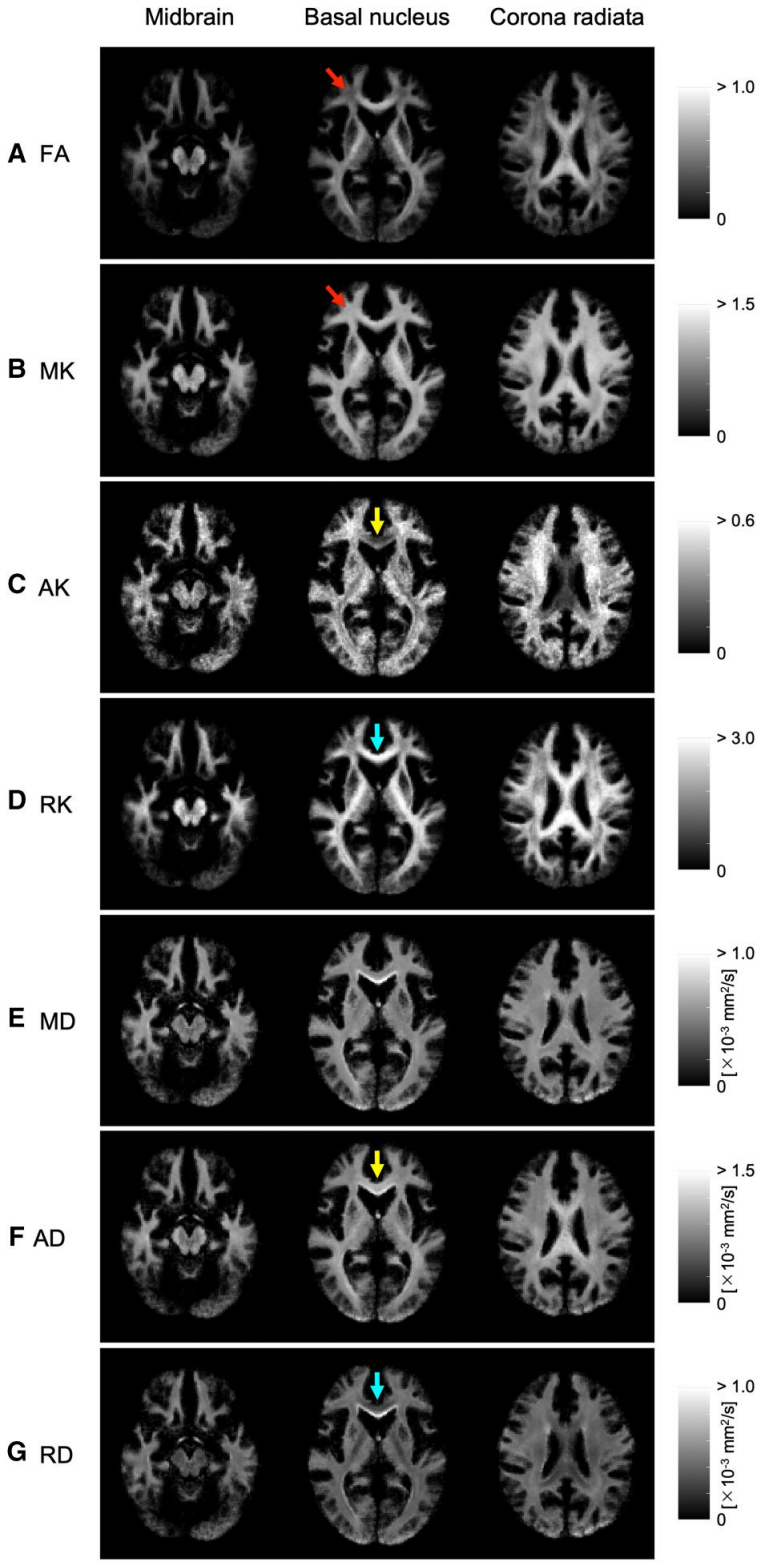
Parameter maps at the midbrain, basal nucleus, and corona radiata levels: A, FA; B, MK; C, AK; D, RK; E, MD; F, AD; G, RD. Figures from left to right show the midbrain, basal nucleus, and corona radiata images. The scale is shown on the right side of each map, and the unit values are shown for diffusivity (×10^−3^ mm^2^/s). Arrows in A and B indicate corona radiata (ACR) areas, and arrows in C, D, F, and G are genu of corpus callosum (GCC) areas. AD, axial diffusivity; AK, axial kurtosis; FA, fractional anisotropy; MD, mean diffusivity; MK, mean kurtosis; RD, radial diffusivity; RK, radial kurtosis.

Figure 3 shows the parameter maps at the midbrain, basal ganglia, and corona radiata levels. Table 1 represents the parameter values in all regions obtained from our experiment: SCC > PLIC > GCC > RLIC > PTR > ALIC > ACR > EC for FA, PLIC > SCC > ALIC > RLIC > ACR > PTR > GCC > EC for MK, ACR > EC > ALIC > PLIC > RLIC > PTR > GCC > SCC for AK, and SCC > PLIC > RLIC > PTR > ALIC > GCC > ACR > EC for RK. For AK, the values in the ACR and EC regions were higher than those in other regions. This fact suggests that the ACR and EC regions are dominated by horizontal components.

**Table 1.** Mean parameter values derived from diffusion kurtosis imaging for each white matter region in thirteen healthy subjects.

Parameters	GCC	SCC	ALIC	PLIC	RLIC	ACR	PTR	EC
FA	0.605 ± 0.275	0.758 ± 0.199	0.576 ± 0.222	0.668 ± 0.193	0.603 ± 0.158	0.467 ± 0.162	0.598 ± 0.197	0.444 ± 0.173
MK	0.919 ± 0.278	1.114 ± 0.188	1.037 ± 0.180	1.164 ± 0.154	1.025 ± 0.136	0.995 ± 0.141	0.952 ± 0.159	0.882 ± 0.130
AK	0.263 ± 0.178	0.244 ± 0.182	0.397 ± 0.240	0.369 ± 0.243	0.349 ± 0.216	0.429 ± 0.225	0.333 ± 0.194	0.405 ± 0.235
RK	1.076 ± 0.495	1.355 ± 0.301	1.121 ± 0.380	1.341 ± 0.290	1.239 ± 0.325	1.005 ± 0.346	1.130 ± 0.373	0.892 ± 0.359
MD	1.029 ± 0.599	0.766 ± 0.260	0.769 ± 0.276	0.701 ± 0.205	0.771 ± 0.222	0.817 ± 0.201	0.855 ± 0.354	0.818 ± 0.193
AD	1.755 ± 0.594	1.632 ± 0.397	1.317 ± 0.359	1.339 ± 0.292	1.350 ± 0.303	1.249 ± 0.258	1.479 ± 0.418	1.223 ± 0.272
RD	0.691 ± 0.641	0.369 ± 0.241	0.529 ± 0.238	0.416 ± 0.181	0.492 ± 0.212	0.603 ± 0.218	0.561 ± 0.355	0.616 ± 0.204

The values of each parameter were measured from 13 healthy subjects data (10 males and 3 females; age range, 21-43 years; mean age, 25.2 years). The values are presented as mean ± standard deviation.

Abbreviations: ACR, anterior corona radiata; AD, axial diffusivity (10^−3^ mm^2^/s); AK, axial kurtosis; ALIC, anterior limb of the internal capsule; EC, external capsule; FA, fractional anisotropy; GCC, genu of corpus callosum; MD, mean diffusivity (10^−3^ mm^2^/s); MK, mean kurtosis; PLIC, posterior limb of the internal capsule; PTR, posterior thalamic radiation; RD, radial diffusivity (10^−3^ mm^2^/s); RK, radial kurtosis; RLIC, retrolenticular part of the internal capsule; SCC, subcallosal cingulate.

**Figure 4.**
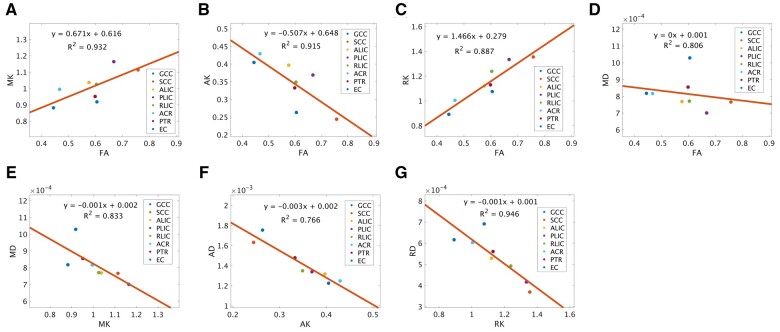
Correlation between DKI and DTI parameter values. A-C, The relationship between FA and DKI parameters (MK, RK, and AK) for WM structures had strong positive correlations for FA-MK, *R*^2^ = 0.932; FA-RK, *R*^2^ = 0.887, and a strong negative correlation for FA-AK, *R*^2^ = 0.915. D) The relationship between FA and MD indicated a negative correlation (*R*^2^ = 0.806). E-G, The relationships between corresponding diffusivity and kurtosis parameters show negative correlations (*R*^2^ = 0.833, 0.766, and 0.946, respectively). AK, axial kurtosis; DKI, diffusion kurtosis imaging; DTI, diffusion tensor imaging; FA, fractional anisotropy; MD, mean diffusivity; MK, mean kurtosis; RD, radial diffusivity; RK, radial kurtosis.

Figure 4 represents the relationship between diffusivity and kurtosis parameters for 13 subjects. Graphs (A), (B), (C), and (D) show results of relationships between FA and MK, FA and AK, FA and RK, and FA and MD, respectively. The strongest correlation coefficient of *R*^2^ = 0.932 can be observed for the relationship between FA and MK. The correlation coefficient between FA and AK is *R*^2^ = 0.915, and this shows a negative correlation. The correlation coefficient between FA and RK is *R*^2^ = 0.887, which is weaker than that of FA and MK. There was observed almost without slope of the linear regression equation between FA and MD (Figure 4D). The relationships between corresponding diffusivity and kurtosis parameters show negative correlations (Figure 4E-G).

**Figure 5.**
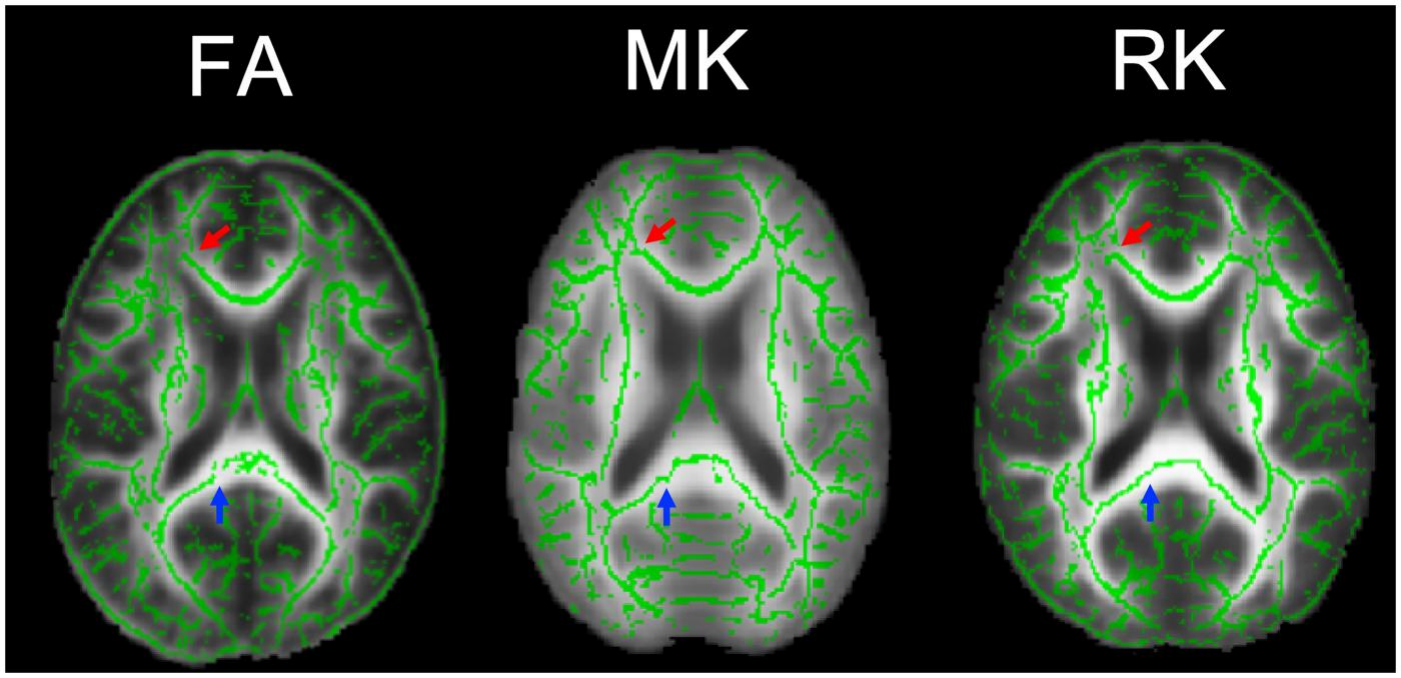
TBSS images of FA, MK, and RK. Upper arrows indicate the anterior corona radiata (ACR) areas, and lower arrows the subcallosal cingulate (SCC) areas. When comparing the TBSS connection in the ACR area, the connection in the MK parameter image could be observed more clearly than those of RK and FA parameters. FA, fractional anisotropy; MK, mean kurtosis; RK, radial kurtosis; TBSS, tract-based spatial statistics.

The images derived from FA, MK, and RK of TBSS are shown in Figure 5. The ACR regions are disconnected when looking at FA and RK images, while corresponding regions are connected in the MK image (Figure 5, red arrows). In the SCC region, the connection between FA was not clearly observed, but MK and RK showed a sufficient connection (Figure 5, blue arrows).

## Discussion

In this study, VBM analysis of DTI and DKI was performed on thirteen healthy subjects, and the corresponding parameter values for each region in the WM template were obtained. DTI and DKI parameter values showed different values in each WM region. There was a small difference in the MD values for each region, but MK values were large (Table 1). The relationship between FA and MK had a stronger positive correlation than that of FA and MD (Figure 4A; *R*^2^ = 0.932), while showing a lower signal in a part of the ACR areas in the FA map (Figure 3A, red arrows). The relationships between MD and MK, between AD and AK, and between RD and RK show negative correlations (Figure 4E-G; *R*^2^ = 0.833, 0.766, 0.946); these parameter maps also had some areas where the contrast was reversed, for example, GCC (Figure 3C and F, arrows; Figure 3D and G, arrows). Our VBM analysis of DKI enabled us to visualize fibre tracts with high signal in structures with multiple fibre crossings, for example, ACR, as well as regions with distribution along parallel fibres, for example, GCC (Figure 5). Thus, DKI parameters may become an evaluation index of normal neuronal structures of myelin and axons in WM tracts.

Das et al^23^ evaluated the age dependence of DKI parameters on the brain in healthy subjects between ages 10 and 79 years. As a result, it was found that there was age dependence in DKI parameters in many regions including GCC and corticospinal tracts, which were found to exist in areas where parameters decreased with age (eg, GCC and EC) and the other areas where parameter values peaked at a certain age (eg, corticospinal tract and body of CC). In other literature,^4^^,^^14^ the order of diffusivity and kurtosis were reported as RD < MD < AD and AK < MK < RK which are the same as our study (Table 1). Because the diffusivity of the axonal direction is calculated as the direction along the pathway of the nerve bundle, the degree of diffusion increases in the axonal direction and the degree of diffusion in the radial direction decreases; therefore, the AD results had the highest value, and RD became the lowest. Kurtosis, which describes the degree of movement restriction of water molecules, is less restricted in the axonal direction and stronger in the radial direction due to the presence of an extremely narrow cell wall with a multi-layered myelin sheath. Thus, in a WM tract, it can be assumed that the relationship RK > AK. In our study, 4 common regions as well as the previous study by Das et al^23^ were measured (eg, GCC, ALIC, PLIC, and EC). In all regions determined in this study, the diffusivities (MD, AK, and RD) were lower than those published in the previous literature, and the RK value for the DKI parameters was higher (Table 1). Although our study was performed with only younger-age subjects, Das et al evaluated subjects over a wide age range including elderly people with decreased myelin. It could be concluded that the RK should have the highest value in DKI parameters. Furthermore, it was found that the DTI parameter has a more considerable difference from the literature value than the DKI parameter. These differences may be caused by technical factors such as the DKI calculation scheme and the imaging parameters (eg, *b*-factors and diffusion directions). Because MK strongly depends on the maximum *b*-value, it should be optimized for both WM and GM.^24^ The maximum *b*-value in our study was 2500 s/mm^2^; our DKI imaging has optimized the effects of the number of *b*-values and diffusion direction on the accuracy of DKI metrics.^16^ In a scanner with a magnetic field strength of 3 T, we estimated that the accuracy of DKI metrics is enabled to maintain; because the accuracy depends on the high signal-to-noise ratio (SNR) of acquired MR data and the dataset with high *b*-values,^16^ and SNR of 3 T is higher than that of 1.5 T scanner unless considerably reducing acquisition time. The study performed by Das et al had a maximum *b*-value of 2000 s/mm^2^. The difference in *b*-values may cause differences between measured and published DTI values. Although it is necessary to overcome such technical factors, DKI can simply describe the degree of deviation from a normal distribution regardless of the biological model assumption. It can be said that the limitations of DTI can be overcome adequately.

The VBM analysis enables semi-automatic morphological analysis of the whole brain, which is less dependent on the analyst's ability. In addition, it leads to the acquisition of information on the differences in the brain shape and volume of the various subjects.^25^ DKI is a relatively simple mathematical model without the assumptions of a biophysical model, which is suitable for clinical application. The VBM analysis is appropriate to intercompare several parameters derived from DTI and/or DKI in the entire brain. The VBM analysis, including the process of standardizing the brain, can perform detailed analysis based on an anatomical template. Thus, VBM analysis using DKI will become a useful analysis tool to clarify not only structural analysis but also pathological structural changes in the brain, by building more databases.

This study has several limitations. First, the number of subjects in this study may not have been sufficient. In our experiment, there was little spatial discrepancy for some regions between the standardized maps and the WM template. Generally, the standardization processing of VBM analysis has been performed for more than 100 subjects. This is because accuracy depends on the number of subjects.^26^ We applied our method to thirteen subjects; the subjects should increase to improve the statistical fluctuations. Second, the subjects in this study were limited to young people whose synapses and myelination had been morphologically completed. In the future, we plan to examine middle-age and elderly age subjects to assess the ageing effect on the DKI parameters. Finally, DWI was performed with the EPI sequence, shortening the scan time. It was found that the magnetic susceptibility caused by the phase dispersion of EPI resulted in image distortion.^27^ In the images acquired in this experiment, image distortion was observed in areas of the surrounding tissue such as the air-containing paranasal sinuses. In our experiment, a distortion correction for each diffusion direction was not performed. When it affects the WM region, B_0_ imhomogeneity correction should be applied^27^ as well as other rigid body transformation algorithms.^28^^,^^29^

## Conclusion

In this study, we evaluate WM structure differences of healthy subjects. Compared with WM analysis using DTI, WM analysis using DKI can provide more detailed information on the connectivity between neural structures. The parameter of DKI, MK, can be used as an index of anisotropic diffusion because of the small variation in numerical values and the strong correlation with FA. This parameter is considered to be most suitable as an evaluation index, because the signal is maintained at the intersections of nerves in TBSS results.
